# Otitis Media Caused by *V. cholerae* O100: A Case Report and Review of the Literature

**DOI:** 10.3389/fmicb.2017.01619

**Published:** 2017-08-28

**Authors:** Peter Kechker, Yigal Senderovich, Shifra Ken-Dror, Sivan Laviad-Shitrit, Eiji Arakawa, Malka Halpern

**Affiliations:** ^1^W. Hirsch Regional Microbiology Laboratory, Clalit Health Services Haifa, Israel; ^2^Department of Evolutionary and Environmental Biology, Faculty of Natural Sciences, University of Haifa Haifa, Israel; ^3^National Institute of Infectious Diseases Toyama, Japan; ^4^Department of Biology and Environment, Faculty of Natural Sciences, University of Haifa at Oranim Tivon, Israel

**Keywords:** *Vibrio cholerae*, otitis media, case report, ear, serogroup identification, antibiogram, virulence genes

## Abstract

Infections due to *Vibrio cholerae* are rarely documented in Israel. Here we report a case of recurrent otitis media in a young male, caused by *V. cholerae* non-O1/O139. This extra-intestinal infection was caused by *V. cholerae* O100 and has been associated with freshwater exposure and travel. Symptoms of chronic periodic earaches along with purulent exudate began about one week after the patient suffered a water skiing accident on a river in Australia. The condition lasted for three years, until his ear exudate was examined in a clinical laboratory, diagnosed and treated. Five bacterial isolates were identified as *V. cholerae* O100. The isolates were screened for genetic characteristics and were found positive for the presence of *hapA*, *hlyA*, and *ompU* virulence genes. All isolates were negative for the presence of *ctxA*. Based on antibiogram susceptibility testing, ciprofloxacin ear drops were used until the patient’s symptoms disappeared. This case demonstrates that exposure to freshwater can cause otitis media by *V. cholerae* non-O1/O139 in young and otherwise healthy humans.

## Introduction

*Vibrio cholerae* is a Gram-negative comma-shaped, facultative anaerobic, motile bacterium, belonging to the *Vibrionaceae* family. *V. cholerae* is both a human pathogen and a natural inhabitant of aquatic environments (Colwell et al., 1977; Cottingham et al., 2003). Infections are primarily associated with ingestion of contaminated foods and water, with subsequent diarrheal illness. This species is divided into more than 200 serogroups, of which only O1 and O139 cause cholera epidemics and pandemics (Harris et al., 2012; Clemens et al., 2017).

*Vibrio cholerae* is a waterborn bacterium and can be found in marine and freshwaters (Clemens et al., 2017). It has been associated with various reservoirs in the aquatic environment: chitinous organisms, i.e., crustaceans and especially copepods (Vezzulli et al., 2010); chironomids (Broza and Halpern, 2001; Halpern et al., 2004, 2006, 2007; Senderovich et al., 2008; Halpern and Senderovich, 2015); phytoplankton and aquatic plants (Islam et al., 2006; Seeligmann et al., 2008); protozoa (Barker and Brown, 1994); waterfowl, which may be the disseminators of *V. cholerae* between water bodies, both within and between continents (Halpern et al., 2008); fish, which consume chironomids and copepods, on one hand, and are consumed by waterfowl on the other, carry *V. cholerae* in their intestine and may play an important role in *V. cholerae* dissemination (Senderovich et al., 2010; Halpern and Izhaki, 2017). Furthermore, in addition to the particle-associated growth strategy of environmental *V. cholerae*, few reports have demonstrated that *V. cholerae* can also grow in water as a free-living organism in the planktonic phase (Worden et al., 2006).

Unlike the O1 and O139 serogroups, *V. cholerae* non-O1/O139 serogroups rarely produce the cholera toxin (CTX), and cause milder gastrointestinal infections, but are associated with extra-intestinal infections, such as septicemia, wound infection, peritonitis, skin infection, cellulitis, necrotizing fasciitis, endophthalmitis, cholecystitis, meningitis and ear infection (Hlady and Klontz, 1996; Centers for Disease Control and Prevention [CDC], 2014; Chen et al., 2015; Hao et al., 2015).

We report here a case of non-O1/O139 *V. cholerae* otitis media which developed in a young male after a water skiing accident.

## Case Report

### Ethics Statement

This study was carried out in accordance with the recommendations of the guidelines of Helsinki Committee and was approved by the Helsinki Committee of Clalit Health Services, Israel (approval no. 0192-15-COM1). The subject gave a written informed consent in accordance with the Declaration of Helsinki.

A 27-years old Caucasian male with acute otitis media presented to an otolaryngologist at a medical clinic in Haifa, Israel, after suffering for a period of three years from a periodic earache accompanied by a malodorous and purulent exudate from his right ear, without fever. As the patient recalled, the onset of the symptoms began approximately one week after his 2011 water skiing accident on the Murray River near Mildura, VIC, Australia, in which he fell and perforated his right eardrum.

Prior to his admission, and with the onset of the periodic ear exudates, the patient had undergone a series of ear cleansings and aspirations without any significant improvement. During his admission, an ear swab was used to sample his exudate, and later was sent to the microbiology laboratory of Clalit Health Services of Haifa and Western Galilee district for further analysis.

Twelve hours after initial incubation a prominent bacterial growth was noticed on Chocolate agar, Tryptic soy blood agar, and MacConkey agar plates (Novamed, Israel). The suspected bacterium was identified as *V. cholerae* by means of VITEK-2 bacterial identifier system (BioMerieux, France) and by using Vitek-MS MALDI-TOF technology (BioMerieux, France). Additionally, five single colonies were picked and streaked on Luria (LB) agar (HiMedia, Mumbai) in five successive days to verify the purity of the isolates; then their identities were verified by a PCR assay in accordance with Nandi et al. (2000). The results demonstrated that all the strains were positive for *ompW*, a gene of an outer membrane protein, specific to *V. cholerae*. The isolate was confirmed as *V. cholerae* non-O1/O139, cholera toxin-negative by the reference laboratory of the Israeli Ministry of Health. Somatic antigen serogrouping identification was employed on the five *V. cholerae* isolates according to Shimada et al. (1994), and all the isolates were identified as *V. cholerae* serogroup O100.

The isolates underwent complete antibiogram susceptibility testing by the disk diffusion technique (Oxoid, United Kingdom) with interpretation according to the *V. cholerae* CLSI guidelines (Clinical and Laboratory Standards Institute [CLSI], 2014). The strains proved resistant to polymyxin B sulfate and susceptible to co-amoxiclav, cefuroxime, co-trimoxazole, gentamicin, and ciprofloxacin. Based on the results of antibiotics activity against the isolates *in vitro*, ciprofloxacin ear drops were used until the patients symptoms disappeared completely. Three weeks later; an additional swab from his right ear was sent to our laboratory; it was found negative for *V. cholerae*.

Furthermore, the presence of virulence genes in the *V. cholerae* isolates was determined. All the isolates were positive for *hapA* (soluble hemagglutinin/protease), *hlyA* (haemolysin), *ompU* (outer membrane protein) and *toxR* (regulatory protein) genes, but negative for the virulence gene cassette such as the cholera toxin gene (*ctxA*), zonula occludens toxin (*zot*), accessory cholera enterotoxin (*ace*) toxin coregulated pilus (*tcpA tcpI*) and for the genes encoding the Type Three Secretion System (TTSS) (*vcsC2*, *vcsN2*, *vspD*, and *vcsV2*). The primers and the PCR procedures for the genes detection are described in Nandi et al. (2000), Dziejman et al. (2005), Halpern et al. (2006), Chatterjee et al. (2009).

## Discussion

*Vibrio cholerae* belongs to the class of environmental pathogens defined as microorganisms that normally spend a substantial part of their life cycle outside the human host but, when introduced into humans, cause disease with measurable frequency (Clemens et al., 2017). While serogroups O1 and O139 cause cholera, the non-O1/O139 serogroups can cause a milder diarrhea, and are also associated with various human infections: blood, wound, ear, and other clinical sites (Dalsgaard et al., 2000).

The patient described in the current case is an immunocompetent young male with no history of chronic ear infections before the water skiing accident in Australia, during which his eardrum was perforated. *V. cholerae* O1 and non-O1/O139 had been isolated from river and marine water over a wide area in Australia since 1977 (Desmarchelier et al., 1988), which may link the ear injury in the Australian river to the subsequent *V. cholerae* otitis media. The water skiing accident happened in January, which is summer in Australia. Water temperatures and *V. cholerae* population size in the aquatic environment usually are higher during this season. Summer months are also the most common months of non-O1/O139 *V. cholerae* infections (Chen et al., 2015).

The isolates identified in this study didn’t harbor the virulence gene cassette such as the cholera toxin gene and the genes encoding Type Three Secretion System. Thus, these genes probably have no significant role in causing otitis media. On the other hand, the isolate harbored other virulence related genes, *hlyA*, *hapA*, *toxR*, and *ompU*. The pathogenic mechanisms of *V*. *cholerae* non-O1/139 in otitis media remain unclear, but these genes might play a role in the disease process. Using cytotoxicity and apoptosis assays, showed that *hly*A-positive strains of *V. cholerae* non-O1*/*O139 had significantly higher cytotoxic activity and levels of apoptosis induction than *hly*A-negative strains, and that a mitochondria-dependent apoptosis pathway is involved (Kanoktippornchai et al., 2014).

Furthermore, *V. cholerae* O100 isolate in the current study was positive to *toxR* and *ompU* genes. ToxR is the master regulator of *V. cholerae* pathogenicity (Faruque et al., 1998). Among other pathogenic genes, it regulates OmpU, one of the major outer membrane proteins of *V. cholerae* (Miller and Mekalanos, 1988). *ompU*, which is an outer membrane porin, has a role in the passage of certain substances through the outer membrane. Also, it has been shown that ToxR controls resistance to various cationic antibacterial proteins, for example, polymyxin B sulfate, through a porin-mediated mechanism (Mathur and Waldor, 2004). Indeed, in the current study, the isolates that were positive to *toxR* and *ompU* genes were resistant to polymyxin B sulfate antibiotics.

Moreover, OmpU is pro-inflammatory in nature. Interestingly, it can also down-regulate LPS (lipopolysaccharide)-mediated proinflammatory responses. OmpU causes suppression of LPS-mediated responses by attenuating the LPS-mediated toll-like receptor signaling pathway (Sakharwade and Mukhopadhaya, 2015). The presence of OmpU protein in the bacterial isolate may explain the phenomenon that was observed in the current case in which the patient suffered from symptoms of earache and purulent exudate for three years. He was cured only when the bacteria was identified and treated with ciprofloxacin ear drops. This antibiotic treatment was effective and all the symptoms completely disappeared.

A few cases of otitis media or otitis externa caused by *V. cholerae* non-O1/O139 are reported in the literature. Most were children (*n* = 7, aged between 9 and 15 years) two young males and one female in their early twenties, and one 49 year- old-man (**Table 1**). The majority of the reported cases were from European countries and only one report was from the United States. In seven cases it was reported that the patients had been swimming in a swimming pool, a lake or in the Baltic Sea.

**Table 1:** Case reports from the literature of otitis caused by *Vibrio cholerae* non-O1/O139 strains.

Case reported	Place	Age years	Gender	Epidemiology	Reference
Protracted middle-ear inflammation	Sweden	10	Male	Bathing in the Baltic archipelago, off Stockholm, *V. cholerae* O107	Back et al., 1974
Middle ear infection	United States	–	–	Exposure to salt water	Hughes et al., 1978
Chronic external otitis	Belgium	12	Female	Unknown	Hansen et al., 1979
Middle ear infection	Romania	–	–	Unknown	Florescu et al., 1981
Otitis media	Belgium	20	Female	Swimming in a swimming pool	Thibaut et al., 1986
External otitis 1 year after eardrum transplant	Belgium	15	Male	Unknown	Thibaut et al., 1986
Otitis media	Germany	–	–	Unknown	Handrick et al., 2004
Chronic recurrent otitis media	Austria	8	Female	Unknown	Huhulescu et al., 2007
Otitis media	Austria	49	Male	Unknown	Huhulescu et al., 2007
Otitis media	Austria	9	Male	Lake Neusiedl in August (year 2004)	Huhulescu et al., 2007
Otitis media	Austria	9	Female	Lake Neusiedl in August (year 2005)	Huhulescu et al., 2007
Otitis externa	Austria	14	Male	Lake Neusiedl in August (year 2000)	Huhulescu et al., 2007
Otitis externa	Austria	22	Male	After vacation in an unknown location	Huhulescu et al., 2007
Ear infection	United Kingdom	–	–	Unknown	Marek et al., 2013
Otitis externa	Austria	21	Female	Swimming in Lake Neusiedl (August, 2015)	Hirk et al., 2016
Otitis media	Israel (Australia)	27	Male	Water skiing accident on the Murray River near Mildura, VIC, Australia (January, 2011) *V. cholerae* O100	The current study

Untreated otitis media can cause complications such as loss of hearing, inner ear and facial nerve problems, including vertigo (Kitsko and Dohar, 2007), and extracranial and intracranial complications (Kangsanarak et al., 1993). Fortunately, the patient in the current case had not such complications. Nevertheless, physicians should be aware that non-O1/O139 *V. cholerae* can cause extra-intestinal infections that can be acquired from exposure to swimming pools and river, lake or marine water. Thus, *V. cholerae* should be added to the differential diagnosis of otitis infections.

## Author Contributions

PK, YS, and SL-S identified the strains. EA, serotyped the strains. SL-S identified the virulence genes. PK and YS performed the antibiogram tests. PK and YS wrote the manuscript, SK-D and MH reviewed the manuscript. MH Edited the manuscript. MH, Contributed reagents/materials/publication fee.

## Conflict of Interest Statement

The authors declare that the research was conducted in the absence of any commercial or financial relationships that could be construed as a potential conflict of interest.
